# Time limit to rescue intestine with viability at risk caused by blood flow disruption in patients presenting with acute abdomen

**DOI:** 10.1007/s00595-022-02495-7

**Published:** 2022-03-25

**Authors:** Takuro Kyuno, Kanki Otsuka, Makoto Kobayashi, Eiji Yoshida, Kei Sato, Ryoko Kawagishi, Tsuyoshi Kono, Takehiro Chiba, Toshimoto Kimura, Hitoshi Yonezawa, Osamu Funato, Akinori Takagane

**Affiliations:** Department of Surgery, Hakodate Goryoukaku Hospital, 38-3, Goryoukaku-cho, Hakodate, Japan

**Keywords:** Strangulation, Incarceration, Volvulus, Intestinal resection, Intestinal rescue rate

## Abstract

**Purpose:**

Early management is crucial for acute intestinal blood flow disorders; however, no published study has identified criteria for the time limit for blood flow resumption. This study specifically examines the time factors for avoiding intestinal resection.

**Methods:**

The subjects of this retrospective cohort study were 125 consecutive patients who underwent emergency surgery for a confirmed diagnosis of intestinal strangulation (*n* = 86), incarceration (*n* = 27), or volvulus (*n* = 12), between January 2015 and March 2021. Intestinal resection was performed when intestinal irreversible changes had occurred even after ischemia was relieved surgically. We analyzed the relationship between the time from computed tomography (CT) imaging to the start of surgery (C-S time) and intestinal resection using the Kaplan–Meier method and calculated the estimated intestinal rescue rate. Patient background factors affecting intestinal resection were also examined.

**Results:**

The time limit for achieving 80% intestinal rescue rate was 200 min in C-S time, and when this exceeded 300 min, the intestinal rescue rate dropped to less than 50%. Multivariate analysis identified the APACHE II score as a significant influencing factor.

**Conclusion:**

A rapid transition from early diagnosis to early surgery is critical for patients with acute abdomen originating from intestinal blood flow disorders. The times from presentation at the hospital to surgery should be reduced further, especially for severe cases.

## Introduction

Accurate diagnosis and prompt initiation of treatment are critical for patients with acute abdomen caused by intestinal ischemia. There are two causes of blood flow obstruction in the intestine: one is an occlusion in the blood vessel caused by a thrombus or embolus, and the other is extravascular pressure such as strangulation, incarceration of a hernia, or volvulus. Generally, if the obstructive mechanism is removed and blood flow in the intestine is released early, intestinal resection for intestinal necrosis is likely avoided. Although the need for prompt initiation of treatment is well recognized, we could find no reports providing specific criteria for the time limit before surgery. Thus, we conducted this study to identify the time factors for avoiding intestinal resection.

## Methods

### Study design

This was a retrospective cohort study of consecutive patients who underwent abdominal emergency surgery in the Hakodate Goryoukaku Hospital between January 2015 and March 2021. The study protocol was approved by the ethics committee of Hakodate Goryoukaku Hospital (Approval No. 2021-042). Baseline data were collected from electronic medical records. The need for informed consent was waived because of the retrospective study design. The committee also confirmed that the data was maintained with confidentiality for the privacy of the patients and was compliant with the Declaration of Helsinki.

### Clinical evaluation and treatment decision

Blood tests and contrast-enhanced computed tomography (CT) scans were performed at the time of the patient’s presentation. The images were double-checked by the surgeon in charge and the diagnostic radiologist. The decision to perform emergency surgery was made by two or more surgical staff members and the anesthesiologist.

### Definitions of data management

The time elapsed from the onset of symptoms to the confirmation of CT diagnosis was defined as the O-C time, and the time from the confirmation of CT diagnosis to the start of surgery was defined as the surgical waiting period (C-S time). The decision criteria for intestinal resection were defined as follows. The decision to resect the diseased intestine was made when the cause of the mechanical blood flow obstruction was surgically removed, the intestinal coloration and intestinal peristalsis did not improve, and the intestinal dysfunction was deemed by two or more surgeons to be irreversible. The Sequential Organ Failure Assessment (SOFA) [1] and Acute Physiology and Chronic Health Evaluation II (APACHE II) [2] were used as tools to assess patient clinical severity before surgery objectively.

### Statistical analysis

Results are presented as means with standard deviation. The student’s *t*-test or Mann–Whitney *U* test was used for univariate analysis. In the time-dependent analysis of the intestinal rescue rate, the C-S time from the first CT diagnosis to the start of surgery was considered the intestinal survival time, and the estimated intestinal rescue rate was calculated by the Kaplan–Meier method. Comparisons were made using the log-rank test. Cox proportional hazard models were used to assess the relationship between the implementation of intestinal resection and the following variables: age, O-C time, SOFA score, APACHE II score, and the preexistence of renal disfunction and diabetes mellitus. Hazard ratios (HRs) and 95% confidence intervals were calculated for all variables. In all analyses, a *P* value < 0.05 was considered to indicate significance. All statistical analyses were performed using IBM SPSS Statistics for Macintosh, version 21.0. (IBM, Armonk, NY).

## Results

### Data collection and management

During the study period, 842 patients underwent emergency surgery for acute abdomen. Among these, 125 patients with a confirmed diagnosis of intestinal strangulation (*n* = 86), incarceration of hernia (*n* = 27), or volvulus (*n* = 12) were included in the present analysis. Among the incarcerations, inguinal and femoral hernias that could be repaired manually were excluded.

### Patient characteristics

Table 1 summarizes the clinical characteristics of the eligible patients. The results of univariate analysis comparing patients with and those without intestinal resection showed significant differences in age, preexisting diabetes mellitus, and the APACHE II score. However, there were no significant differences in postoperative complications between the groups. Notably, there was one patient in the intestinal preservation group who underwent reoperation for postoperative perforation but subsequently died of septic disseminated intravascular coagulation (DIC) and multiple organ failure. Additionally, postoperative survival measured by hospital days to discharge was significantly prolonged in the intestinal resection group.

**Table 1:** Clinical characteristics of the patients with and those without intestinal resection. *SD* standard deviation, *SOFA* sequential organ failure assessment, *APACHE II* acute physiology and chronic health evaluation II, *CT* computed tomography, *SSI* surgical site infection, *DIC* disseminated intravascular coagulation

Clinical characteristics	Intestinal resection		*P*-value
	Yes	No	
Patients, *n*	83	42	
Age, mean (SD)	77 (10.8)	68 (16.2)	< 0.01
Sex, males (%)	33 (40)	16 (38)	1.00
Diagnosis, *n*			0.14
Intestinal strangulation	59	27	
Incarceration	14	13	
Volvulus	10	2	
Comorbidity, *n*			
Hypertension	41	17	0.45
Diabetes mellitus	24	3	< 0.01
Chronic kidney disease	6	2	0.72
SOFA score, *n* (%)			0.051
≦3	67 (81)	40 (95)	
≧4	16 (19)	2 (5)	
Mean (SD)	2.0 (1.7)	1.4 (1.4)	
APACHE II score, *n* (%)			0.042
≦11	48 (58)	31 (74)	
≧12	35 (42)	11 (26)	
Mean (SD)	11.4 (4.5)	9.6 (4.3)	
Time from onset to surgery (min), *n*			0.24
< 1440	44	25	
1440–2879	13	10	
2880–4329	10	3	
≧4320	16	4	
Mean (SD)	2910 (3688)	2128 (2949)	
Time from onset to CT (min), *n*			0.53
< 1440	52	30	
1440–2879	13	5	
2880–4319	4	4	
≧4320	14	3	
Mean (SD)	2166 (2900)	1815 (2961)	
Time from CT to surgery (min), *n*			0.22
< 180	12	11	
180–299	38	22	
300–419	10	3	
≧420	23	6	
Mean (SD)	743 (2139)	328 (3326)	
Postoperative complications, *n*			
SSI	7	0	0.094
Ileus	13	5	0.79
Aspiration pneumonia	7	2	0.72
Sepsis / DIC	6	1	0.42
Perforation (re-operation)	1	1	1.00
Outcome, *n* (%)			0.66
Survived	79 (95)	41 (98)	
Died in hospital	4 (5)	1 (2)	
Postoperative survival hospital days, mean (SD)	16.5 (12.4)	11.2 (9.4)	0.016
Pathological findings, *n* (%)			
Changes in all layers, necrosis or perforation	77 (93)	–	
Mucosal/submucosal changes	6 (7)	–	

### Intestinal rescue rate and affecting factors

**Fig. 1:**
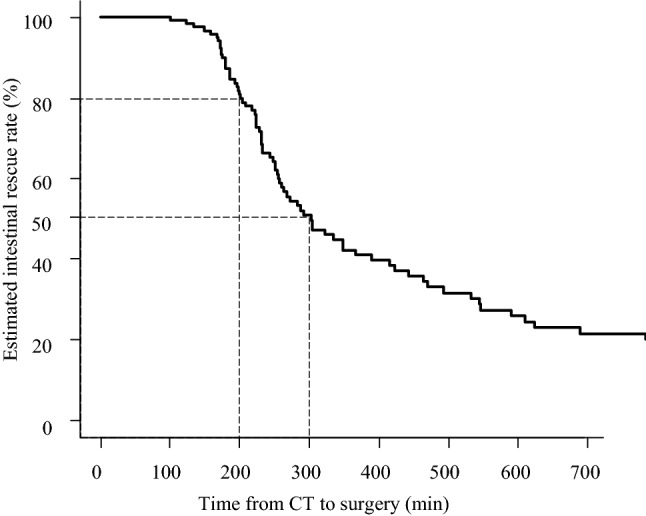
Estimated intestinal rescue rate curve on the elapsed time from the confirmation of the computed tomography diagnosis to the start of surgery (C-S time). The time limit for achieving 80% intestinal rescue rate was 200 min of C-S time, and the intestinal rescue rate decreased to less than 50% when the C-S time was over 300 min. *CT* computed tomography, *C-*S time the elapsed time from the confirmation of CT diagnosis to the start of surgery

**Fig. 2:**
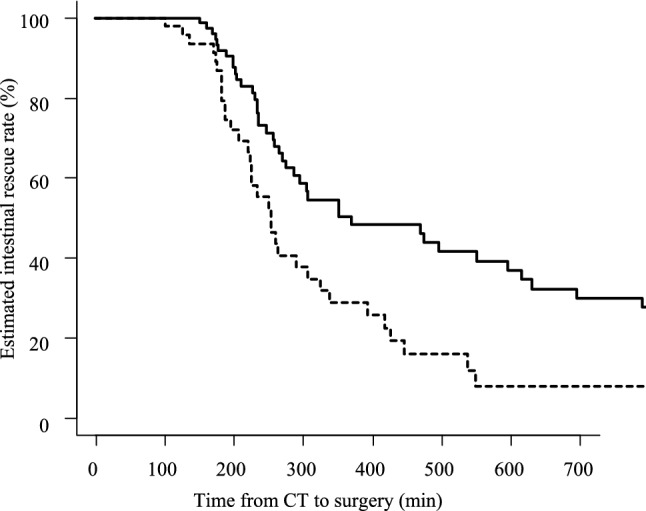
Comparison of the estimated intestinal rescue rate in the patients with an APACHE II score < 12 and those with an APACHE II score ≥ 12. This figure illustrates the comparative findings between patients with APACHE II score < 12 (*n* = 79, solid line) and those with an APACHE II score  ≥ 12 (*n* = 46, dotted line). The intestinal rescue rate differed significantly between the two groups by log-rank test (*P* = 0.0031). *APACHE II* Acute Physiology and Chronic Health Evaluation II, *CT* computed tomography, *C-S *time the elapsed time from the confirmation of CT diagnosis to the start of surgery

**Fig. 3:**
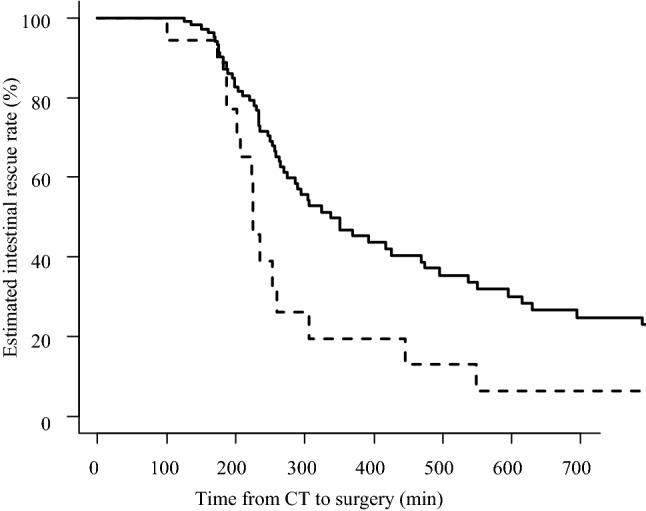
Comparison of the estimated intestinal rescue rate between patients with a sequential organ failure assessment (SOFA) score ≤ 3 and those with a SOFA score ≥ 4. This figure illustrates the comparative findings between patients with a SOFA score ≤ 3 (*n* = 107, solid line) and those with a SOFA score ≥ 4 (*n* = 18, dotted line). The intestinal rescue rate differed significantly between the two groups by log-rank test (*P* = 0.0048). *SOFA* Sequential Organ Failure Assessment, *CT* computed tomography, *C-S time* time elapsed from the confirmation of CT diagnosis to the start of surgery

Figure 1 shows the estimated intestinal rescue rate. The time limit for achieving 80% intestinal rescue rate was 200 min of C-S time, and when this exceeded 300 min, the intestinal rescue rate dropped to less than 50%. A multivariate analysis of factors affecting the intestinal resection rate pointed to a significant effect of the APACHE II score (HR: 1.070, *P* = 0.028), but did not reveal an effect of the O-S time (HR: 1.000, *P* = 0.67). To evaluate the relationship between preoperative clinical condition and intestinal rescue rate, we examined the effect of the APACHE II and SOFA scores on the intestinal rescue rate. Using a Cox proportional hazards model, we found that an APACHE II score of 12 points had the greatest HR (HR: 1.94, *P* = 0.0037). Therefore, we divided the eligible patients into two groups: those with APACHE II scores of ≤ 11 [*n* = 79] and those with APACHE II scores of ≥ 12 [*n* = 46], and examined them via Kaplan–Meier analysis. We found that those with an APACHE II score of ≥ 12 had a significantly lower intestinal rescue rate (Fig. 2). On the other hand, a SOFA score of 4 points was selected as the borderline value (HR: 2.18, *P* = 0.0060). Similarly, we divided the patients into two groups: those with SOFA scores of ≤ 3 [*n* = 107] and those with SOFA scores of ≥ 4 [*n* = 18], and examined them via Kaplan–Meier analysis. We found a significant decrease in the intestinal rescue rate of patients with SOFA scores ≥ 4 (*P* = 0.0048, Fig. 3).

### Histopathological examination of resected intestines

Table 1 shows the findings of histopathological analysis of intestines resected based on intraoperative judgment. The tissue changes were confined to the mucosal or submucosal layer in 6 (7.2%) patients, whereas damage was evident in all layers of the intestine or resulted in necrosis or perforation in 77 patients (92.8%).

## Discussion

Acute abdomen is a general term for the sudden onset of severe abdominal pain that is likely to require emergency surgery and is caused by a wide variety of conditions [3]. There is a lack of knowledge about the impact of early intervention on outcomes and we could find no reports examining the time limits for emergency surgery to be initiated. In this study, we examined the time limit from diagnosis to surgery for conditions in which the disturbance of blood flow in the intestine can be corrected by surgical manipulation. As a result, we concluded that surgery should be started within 200 min of confirmation of the diagnosis to achieve an 80% intestinal rescue rate.

The disruption of blood flow in the intestine leading to ischemia should be restored before it transitions to irreversible intestinal unviability [4, 5]. The causes of impaired intestinal blood flow include superior mesenteric artery occlusion (SMAO), non-occlusive mesenteric ischemia (NOMI), and portal vein occlusion, which are caused by intravascular obstruction. In contrast, strangulation, incarceration, and volvulus are conditions that can be caused by an extravascular mechanism obstructing intestinal vessels. Contrast-enhanced CT scans are important in the diagnosis of blood flow disorders [6]. Findings such as poor contrast enhancement, thickening or thinning of the intestinal wall, abrupt change in the intestinal diameter, intestinal emphysema, and portal vein gas suggest disorders of intestinal blood flow [7–10]. In terms of treatment, interventional radiology (IVR) has become increasingly useful for vascular occlusions caused by thrombus or embolus [11, 12]. Surgical treatment is indicated when the occlusive mechanism was extravascular. The progression to irreversible changes may vary depending on whether the blood flow obstruction is arterial or venous, and whether the occlusion is complete or incomplete. It is difficult to define how much and for how long an organ can withstand ischemia [13], but there is no disagreement about the importance of initiating treatment as soon as possible.

When considering the time factor from diagnosis to the start of treatment, the time from the onset of symptoms to a presentation at a hospital in a real situation depends on the patient's behavior. From the time the patient arrives at the hospital, the process of medical treatment is defined by the response of the hospital staff. If emergency surgery is required, the process that can be shortened involves the time from diagnosis to the start of surgery. An accurate knowledge of the impact of time reduction on treatment outcomes is useful for reviewing the clinical process. For example, the importance of the time limit from the onset of cerebral infarction and ischemic heart disease to the start of treatment is already well known in emergency medicine [14, 15], but there are no specific guidelines for the time limit in intestinal blood flow disorders. In the present series, the hospital stay of patients who underwent intestinal resection was prolonged, and it was clear that the avoidance of intestinal resection would be of great benefit to these patients. Therefore, we believe that this study is clinically meaningful because it establishes the time limit between the confirmation of diagnosis and the start of surgery.

For this analysis, we chose intestinal strangulation, incarceration, and volvulus as target pathologies because a definitive treatment can be initiated to restore blood flow and intestinal viability can be evaluated during the operation. On the other hand, IVR is prioritized as the treatment for SMAO and NOMI caused by intravascular disorders; however, it is difficult to assess whether intestinal viability has been recovered at the time of treatment [11, 12]. Incarceration of inguinal and femoral hernias is subject to emergency surgery if the manual reduction is unsuccessful, but when the incarceration can be reduced manually, strict follow-up is performed as the condition of the intestine is not directly confirmed by surgery; therefore, these cases were excluded from the analysis.

In addition to the effect of the time of starting surgery, the O-C time, the severity of the patient's illness before surgery, and the presence of complications may also influence the viability of the intestine. The Cox hazard analysis of covariates that might affect the intestinal survival rate showed that only the preoperative APACHE II score remained a strongly influential factor. On comparing the APACHE II in the two groups divided by a score of 12, the estimated curve of the intestinal rescue rate showed a significant leftward shift in the severe group with a score of 12 or higher (Fig. 2). Similarly, in the two groups classified by SOFA score, the curve was shifted to the left in the group with a SOFA score ≥ 4 (Fig. 3). These results demonstrate that patients with the poor systemic condition need surgery earlier. On the other hand, the presence of preoperative complications and the length of O-C time did not affect the intestinal rescue rate. This means that fatal intestinal blood flow disturbance starts when the patient presents in unbearable pain and the C-S time is the most important time factor affecting intestinal viability.

The present study has several limitations. First, it was a retrospective study conducted in a single institution without randomization, and the study cohort was small and collected over a 6-year period. Second, there is also a limitation in terms of the validity of the decision to perform intestinal resection. Histopathological examination of the resected intestines showed that ischemic changes were limited to the mucosa or submucosa in 7% of cases and these changes could have been prevented by removing the cause of ischemia during surgery. Therefore, a highly objective evaluation method is needed to determine the need for intraoperative intestinal resection. Recently, the fluorescence method using indocyanine green for the intraoperative evaluation of intestinal blood perfusion has been used to prevent anastomotic leakage during gastrointestinal surgery, especially colorectal surgery [16, 17]. This method has also been used for evaluating intestinal ischemia [18–23], allowing surgeons to identify the extent of ischemia objectively by visualizing the blood supply to the intestine. This method can potentially minimize the extent of intestinal sacrifice. Third, in clinical practice, it is assumed that the intestine may already be in an irreversible state before the start of surgery, and the true rescue time may be shorter than the results of the estimated intestinal rescue rate in this paper. Fourth, the availability of operating rooms, anesthesiologists, and surgical staff, and various administrative procedures can be the rate-limiting factors before the start of surgery. To address these issues, the entire hospital organization needs to establish a system to shorten the interval between presentation and surgery required for emergencies.

In conclusion, intestinal resection should be avoided as it prolongs hospital stay and is detrimental to the patient. According to the results of the present analysis, an 80% intestinal rescue rate can be achieved if intestinal blood flow can be restored within 200 min after diagnosis, but the rescue rate drops to less than 50% after 300 min. In acute abdomen originating from intestinal blood flow disorders, it is necessary to strive for a smooth and shorter transition from early diagnosis to early surgery, especially in severe cases.
